# Transmission of *Schistosoma mansoni* in Yachi areas, southwestern Ethiopia: new foci

**DOI:** 10.1186/s40249-018-0513-5

**Published:** 2019-01-10

**Authors:** Teshome Bekana, Wei Hu, Song Liang, Berhanu Erko

**Affiliations:** 1 Aklilu Lemma Institute of Pathobiology, Addis Ababa University, Addis Ababa, Ethiopia; 2 Department of Biomedical Science, Faculty of Public Health and Medical Science, Mettu University, Mettu, Ethiopia; 3 Department of Microbiology and Microbial Engineering, School of Life Science, Fudan University, Shanghai, China; 4 Department of Environmental and Global Health, College of Public Health and Health Professions, University of Florida, Gainesville, FL 32610 USA

**Keywords:** *Schistosoma mansoni*, Transmission foci, Prevalence, School children, Yachi areas, Ethiopia

## Abstract

**Background:**

*Schistosoma mansoni*, causing intestinal schistosomiasis, is widely distributed in Ethiopia and new transmission foci are continually reported. Here we report new transmission sites and prevalence of *S.mansoni* infection among school children in Yachi areas, southwestern Ethiopia.

**Methods:**

A cross-sectional survey was conducted among school children of Yachi Yisa and Yachi Efo elementary schools, southwestern Ethiopia, from April 2017 to June 2017. Three hundred seventeen school children aged six to 15 years were randomly selected to provide stool specimens for helminth infection examination by Kato-Katz and formol-ether concentration techniques. Snail survey was carried out to assess schistosome infection in *Biomphalaria pfeifferi.* Laboratory bred mice were also exposed to schistosome cercariae shed by *B. pfeifferi en masse* for definite identification of *Schistosoma* species.

**Results:**

From the 317 stool specimens examined using double Kato-Katz thick smear and single formol-ether concentration techniques, 224 (70.7%) were found positive for at least one intestinal helminth species. The most prevalent parasite was *S. mansoni* (42.9%) followed by *Trichuris trichiura* (34.1%) and *Ascaris lumbricoides* (14.2%). The prevalence of *S. mansoni* infection was significantly higher among the children attending Yachi Yisa School (49.4%) than those in Yachi Efo School (35.6%) (*P* = 0.002). The study also revealed that there was a significantly higher prevalence of *S.mansoni* infection among males (51.2%) than females (33.1%) (*P* < 0.001). However*,* the prevalence of *S.mansoni* infection was not significantly associated with age categories (*P* = 0.839). *B. pfeifferi* snails infected with schistosomes were collected from the water bodies found in the study area. After six weeks post exposure, adult *S. mansoni* worms were harvested from the mesenteric veins of laboratory bred mice.

**Conclusions:**

The study revealed establishment of new *S. mansoni* transmission foci and moderate prevalence of schistosomiasis in Yachi areas. Hence, treatment of all school-age children once every two years is recommended. Snail control and non-specific control approaches including provision of clean water supply and health education should also complement mass drug administration of praziquantel.

**Electronic supplementary material:**

The online version of this article (10.1186/s40249-018-0513-5) contains supplementary material, which is available to authorized users.

## Multilingual abstracts

Please see Additional file 1 for translations of the abstract into the five official working languages of the United Nations.

## Background

Schistosomiasis, a major parasitic disease due to infection by *Schistosoma mansoni*, S*. haematobium*, *S. japonicum*, *S. mekongi, S. intercalatum*, and *S. guineensis*, is estimated to affect more than 240 million people worldwide, with 700 million people being at risk of infection [1]. Infection with *S. mansoni* and *S. haematobium* is predominant in tropical and subtropical countries, particularly in sub-Saharan Africa (SSA) [2]. The estimated disability-adjusted life years lost due to the disease was 1.9 million in 2016 [3]. It is a major public health problem and affects people living in rural areas where poor personal hygiene, lack of access to clean water and inappropriate disposal of human faeces and urine enhance transmission. The disease spreads especially in freshwater environment where specific intermediate snail hosts for the parasite are flourishing and where people come into contact with water containing cercariae [2]. School age children are particularly vulnerable to infection due to their frequent contact with infected water and low levels of acquired immunity [4]. In infected children the disease has been associated with stunted growth, anemia and poor cognitive performance [5].

In Ethiopia schistosomiasis is one of the prevalent parasitic diseases reported across many regions, causing considerable morbidity, with over five million people estimated to be infected, and more than 37 million people to be at the risk of infection [4]. The intestinal schistosomiasis caused by *S.mansoni* is present in most parts of the country, whereas the urogenital form caused by *S. haematobium* is found in some low land areas [4]. Epidemiological studies conducted since 1961 have shown that the disease is spreading in connection with a range of socio-economic factors including increased population density and movements, as well as ecological change and environmental modifications resulting from extensive water resources development [6–8].

According to reports from health centers in Yachi areas, *S.mansoni* and soil transmitted helminth (STH) infections are major public health problems reported in communities. Despite the frequent case reports from local health offices and the presence of suitable environment for snail reproduction, studies reporting the establishment of schistosomiasis transmission is lacking in the area. Therefore, this study was conducted to determine the transmission and prevalence of *S. mansoni* infection in Yachi areas, Guma and Gomma districts, southwestern Ethiopia.

## Methods

### Study site

**Fig. 1:**
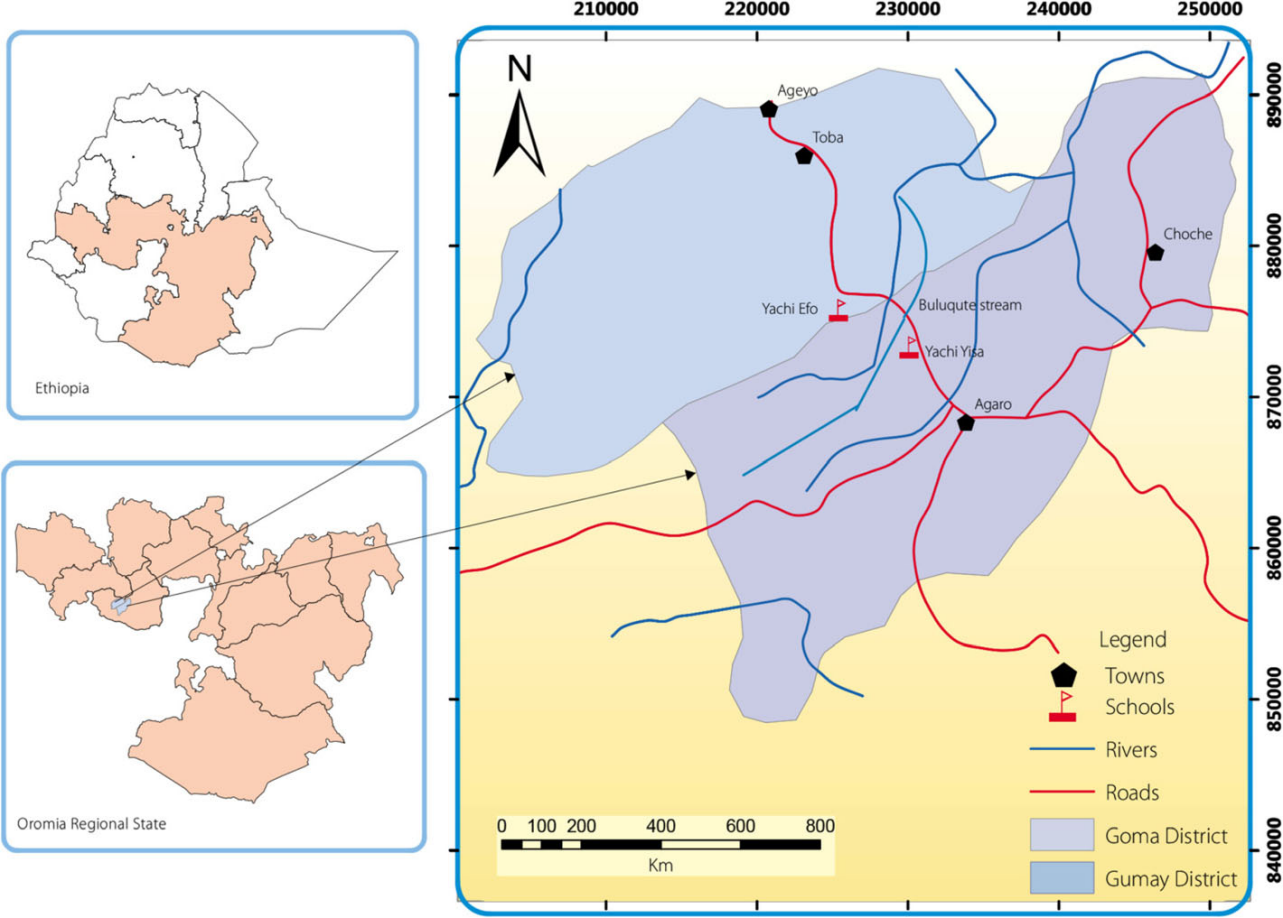
Map showing the study area, southwestern, Ethiopia

The study was conducted in Yachi Efo Elementary School of Guma District and Yachi Yisa Elementary School of Gomma District, two adjacent districts of Jimma Zone, southwestern Ethiopia. The area is located 412 km to the southwest of Addis Ababa. The schools lie between longitude 36°30*′*E and 36°31*′*E and latitude 7°25*′*N and 7°58*′*N, at an altitude of 1728 m above sea level. The area is characterized by warm climate with daily mean temperature of 13.4–27 °C and annual mean rainfall of about 1850 mm (Fig. 1). It has a dry season in the winter (from November to February) and rainy season in the summer (from March to October). Many water bodies including, Yachi River, Laga Jawe River, Buluqute and Yamo streams traverse the study area. These water bodies are widely used for small scale irrigation and for domestic purposes by the local inhabitants, and are identified as suitable environments for the thriving of snail intermediate hosts. *Khat* (*Catha edulis*) and coffee (*Coffea arabica L*.) are the predominant cash crops pruduced by the local inhabitants living in the area.

### Study design and sample size determination

A cross-sectional parasitological survey was conducted among school children of Yachi Yisa and Yachi Efo elementary schools in southwestern Ethiopia, from April 2017 to June 2017. The sample size required for the study was calculated using the single population proportion formula $n=\frac{Z^2\left(1-\frac{\alpha }{2}\right)P\left(1-P\right)}{d^2}$, with the consideration of prevalence (p) of 24.0% from previous study conducted in Jimma Zone [9], Z (1-α/2) = 1.96 at 95% confidence interval (*CI*) and precision value (d) of 5%. Assuming a non-response rate of ~ 20%, the sample size was estimated to be 336 (rounded to 332).

### Sampling technique

Yachi Efo and Yachi Yisa elementary schools were purposely selected because they are located in close proximity to small water bodies and perennial streams, which create suitable habitat for snail intermediate hosts. Children and principal teachers were informed about the aim and procedures of the study. A list of children aged six to 15 years (from grade 1 to grade 5) was obtained from the class registers of both schools. The calculated sample size (332) was then proportionally allocated to Yachi Yisa (175) and Yachi Efo (157) elementary schools. The study participants were selected by systematic sampling technique using class registers as the sampling frame. Of the selected children, 317 (88.8%) who met the inclusion criteria (obtaied written signed consent and provided sufficient stool specimens) were included in the final analysis.

### Field and laboratory procedures

To collect stool specimens, the selected children were given clean plastic sheet with wooden applicator stick and instructed to provide their own feces (approximately 5 g). Two Kato-Katz (KK) slides (using 41.7 mg template) per stool were prepared in the field and the remaining portion of stool specimens (approximately 3 g) were preserved in 10% formalin solution for examination by formol-ether concentration technique (FECT). The prepared slides and preserved specimens were transported to the Medical Parasitology Laboratory of the Aklilu Lemma Institute of Pathobiology, Addis Ababa University, within four to five days of specimen collection. Quantitative examination for *S. mansoni, Ascaris lumbricoides*, *Trichuris trichiura*, *Fasciola* species, and qualitative examination for *Enterobius vermicularis, Strongyloides stercoralis, Hymenolepis nana* and *Taenia* species were done in the laboratory within two weeks of specimen delivery. The KK thick smear technique was used for the qualitative examination and the quantification of *S. mansoni* eggs and the detection of other intestinal helminth species. The intensity (expressed in eggs of per gram of stool, EPG) of *S. mansoni* infection was determined by multiplying the mean of eggs counted in both KK slides by a factor of 24 [10]. Examination of the stool specimens for hookworm eggs was not done in the field due to the unavailability of electric power in the area. The preserved specimens were processed using FECT for the detection of *S. mansoni* and other intestinal helminth infections [10]. A stool specimen from each person was considered positive for *S. mansoni* or other intestinal helminth species if egg or larvae were detected by either of the two or both methods. A specimen was considered negative for *S. mansoni* or other intestinal helminth species if no egg or larvae of these parasites were detected by KK thick smear technique and FECT. Infection intensity of *S. mansoni* was categorized according to WHO criteria [11] as light (1–99 EPG), moderate (100–399 EPG) and heavy (> 400 EPG). For quality control purpose, 10% of the KK slides were randomly selected and re-examined by an experienced microscopist who was blind to the original results.

### Snail survey

Surveys of snails from water contact sites in the water bodies were carried out to determine schistosome infection in the snails. The snails were collected during wet season in 2017 (April to May) by using a wire mesh scoop attached to a wooden handle. The collected snails were then placed in pre-labelled plastic buckets containing water and aquatic weeds, and transported to the Medical Parasitology Lab of Aklilu Lemma Institute of Pathobiology. Before the determination of infection, the collected snails were identified to species level based on shell morphology [12]. *B. pfeifferi* snails varying in diameter from 8 mm to 12 mm were placed in group of ten in Petri dish containing aged water and exposed to indirect sunlight for 40 min to stimulate cercarial shedding.

### Mice infection

Ten laboratory-bred Swiss albino mice aged four to five months and free from previous *S. mansoni* infection were exposed to the schistosome cercariae shed from the *B. pfeifferi* snails. Mice were exposed by immersing legs and tails of each mouse in a beaker containing cercariae mixed in 10 ml of aged water (tap water allowed to age for at leat 24 h prior to use) for 30 min. Fecal specimens from the mice were examined for the presence of *S. mansoni* eggs starting from day 40 post exposure. After six weeks post-exposure, all mice were scarified and the number of schistosomes in the mesenteric veins was counted [13].

### Data analysis

Data were entered into Microsoft Excel 2007 spreadsheet (Microsoft corporation, Redmond) and analysed with SPSS version 20.0 (IBM Corporation, New York, USA). Bivariate and multivariable logistic regression analyses were used to test statistical difference in prevalence of *S. mansoni* infection between schools, sex and age-groups. Differences in infection intensity of *S. mansoni* between groups were tested using the student’s *t*-test. *P* values of < 0.05 were considered statistically significant.

## Results

**Fig. 2:**
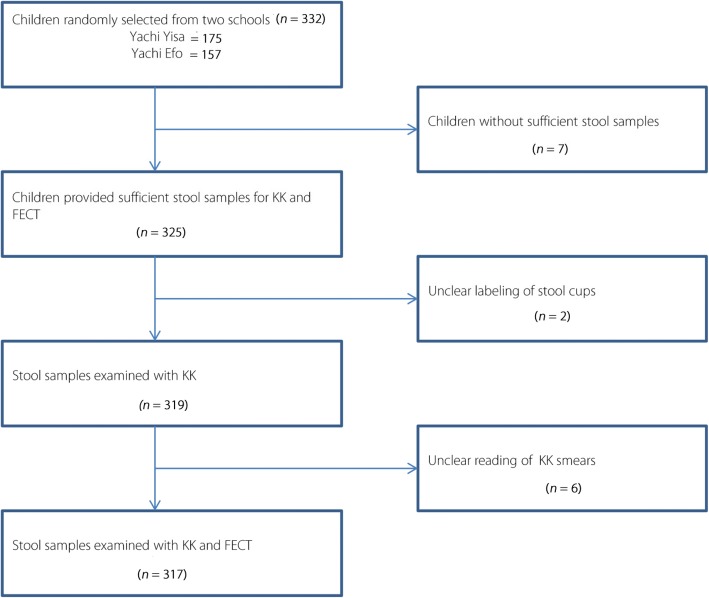
Flow chart detailing the study design. KK: Kato-Katz, FECT: Formol ether concentration technique

A total of 317 (95.5%) school children out of 332 selected provided sufficient stool specimens for examination by KK thick smear technique and FECT. The mean age of children was 10.1 years (Range: 6–15 years, 95% *CI*: 6.8–13.4) and 172 (54.3%) of the children were males. One hundred forty nine (47.1%) were from Yachi Efo School and 168 (52.9%) were from Yachi Yisa School (Fig. 2).

### Prevalence of *S. mansoni* and other intestinal helminth infections

**Fig. 3:**
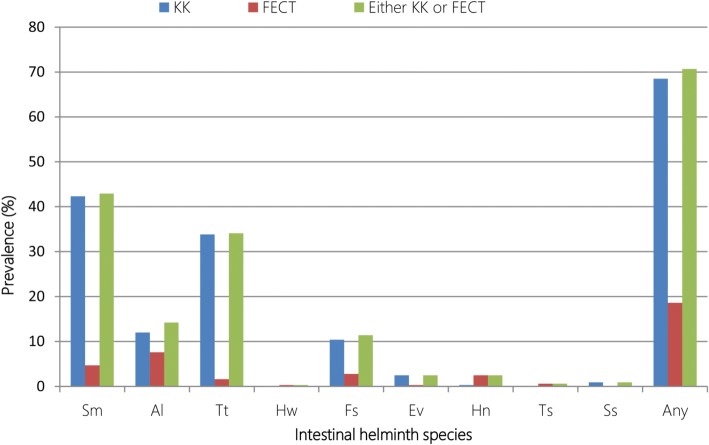
Prevalence of intestinal helminth infections among school children in Yachi areas, southwestern Ethiopia, 2017. KK: Kato-Katz, FECT: Formol ether concentration technique, Sm: *S. mansoni*, Al: *Ascaris lumbricoides*, Tt: *Trichuris trichiura*, Hw: Hookworm species, Fs: *Fasciola* species, Ev: *Enterobius vermicularis*, Hn: *Hymenolepis nana*, Ts: *Taenia* species, Ss: *Strongyloides stercoralis*, Any: infected with at least one intestinal helminth species

Out of 317 stool specimens examined using double KK smears and single FECT, 224 (70.4%) were found positive for at least one intestinal helminth species. Nine species of intestinal helminth parasites were identified in this study. The prevalent intestinal helminth detected were *S. mansoni* (42.9%), *T. trichiura* (34.1%) and *A. lumbricoides* (14.2%) and (11.4%) while the rare parasites detected were *Fasciola* species, *H. nana*, *E. vermicularis*, *S. stercoralis*, *Taenia* species, and hookworms (Fig. 3).

Of the 317 stool specimens examined, 68.7% (217), 18.6% (59) and 70.7% (224) were diagnosed positive for intestinal helminth infections when examined by double KK smear, FECT and by either KK or FECT techniques, respectively. Out the stool specimens positive for *S. mansoni* infection, 136 (42.9%) were positive by KK thick smear technique, while 15 (4.7%) were positive only by FECT. The prevalence of multiple helminth infections obtained with both methods were 20.5% (65/317), 6.3% (20/317) and 0.3% (1/317) for double, triple and quadruple, respectively (Fig. 3).

Bivariate analysis showed that sex (c*OR* = 0.5, 95% *CI*: 0.3–0.7); *P* = 0.001) and school (c*OR* = 0.6, 95% *CI*: 0.4–0.9; *P* = 0.013) were significantly associated with prevalence of *S. mansoni* infection. The prevalence of *S. mansoni* infection was slightly higher among children aged 11–15 years (44.4%) compared to those aged 6–10 years (41.6%), but the difference was not statistically significant (c*OR* = 1.1, 95% *CI*: 0.7–1.7; *P* = 0.731). In the multivariable logistic regression analysis, sex (male) (a*OR* = 0.4, 95% *CI*: 0.2–0.6; *P* < 0.001) and school (Yachi Yisa) (a*OR* = 0.4, 95% *CI*: 0.2–0.7; *P* < 0.002) were found to be associated with the prevalence of *S. mansoni* infection (Table 1).

**Table 1:** Prevalence and mean intensity of *S. mansoni* infections by age, sex and school among school children in Yachi areas, southwestern Ethiopia, 2017

Variable		Frequency *of Schistosoma mansoni*		Bivariate		Multivariate		ARMEC ^b^	
	*N*	Positive	Prevalence (%)^a^ (95% *CI*)	c*OR* (95% *CI*)	*P* value	a*OR* (95% *CI*)	*P* value		*P* value
Overall	317	136	42.9 (37.4–0.48.3)					760.2	
Age (years)									
5–10	173	72	41.6 (34.2–48.9)	1		1		178.3	0.477^c^
11–15	144	64	44.4 (36.3–52.5)	1.1 (0.7–1.7)	0.731	0.9 (0.6–1.6)	0.839	141.1	
Sex									
Male	172	88	51.2 (43.7–58.6)	0.5 (0.3–0.7)	0.001	0.4 (0.2–0.6)	< 0.001	220.8	0.014^c^
Female	145	48	33.1 (25.4–40.7)	1		1		95.3	
School									
Yachi Efo	149	53	35.6 (27.9–43.3)	1		1		93.7	0.010^c^
Yachi Yisa	168	83	49.4 (41.8–56.9)	0.6 (0.4–0.9)	0.013	0.4 (0.2–0.7)	0.002	225.3	

Abbreviations: a*OR*: Adjusted odds ratio, ARMEC: Arithmetic mean of egg count, *CI*: Confidence interval, c*OR*: Crude odds ratio, ^a^ determined byboth methods, ^b^ determined by Kato-Katz, ^c^ Obtained by student’s *t*-test, *N*: Number examined

### Intensity of *S. mansoni* infection

The overall arithmetic mean egg count (ARMEC) for *S. mansoni* was 760.2 (ranging from 24 to 4500 EPG). Table 2 presents the prevalence of heavy, moderate and light infection intensity of *S. mansoni*. Overall, 11.7% (6.3–17.9); 10.7% (5.0–15.8) and 20.5% (13.7–27.3) of the children had heavy, moderate and light infection intensity of *S. mansoni*, respectively. Males had significantly higher intensities of infection than females (*t* = 2.46, *P* = 0.014). Infection intensity of *S. mansoni* was significantly higher among the children attending Yachi Yisa School (225.3 EPG) compared to those in Yachi Efo School (93.7 EPG) (*t* = 2.59, *P* = 0.010). However*,* intensity of *S. mansoni* infection was not significantly associated with age categories(*t* = 0.71, *P* = 0.477) (Table 1).

**Table 2:** Prevalence (%) of heavy, moderate and light *S. mansoni* infection

Category	Classification of intensity of *Schistosoma mansoni* infection		
	Heavy (95% *CI*)	Moderate (95% *CI*)	Light (95% *CI*)
Overall	11.7 (6.3–17.9)	10.7 (5.0–15.8)	20.5 (13.7–27.3)
Age (years)			
5–10	17.6 (11.2–24.0)	13.2 (7.5–18.9)	30.1 (22.4–37.8)
11–15	9.6 (4.6–14.5)	11.8 (6.4–17.2)	17.6 (11.2–23.9)
Sex			
Male	20.6 (13.8–27.4)	16.9 (10.6–23.2)	27.2 (19.7–34.7)
Female	6.6 (2.4–10.8)	8.1 (3.5–12.7)	20.6 (13.8–27.4)
School			
Yachi Efo	5.9 (1.9–9.8)	13.2 (7.5–18.9)	19.8 (13.1–26.5)
Yachi Yisa	21.3 (14.4–28.2)	11.8 (6.4–17.2)	27.9 (20.4–35.4)

### Snail infection

The two snail species identified from the Buluqute and Yamo streams in the study area were *B. pfeifferi* and *Lymnaea natalensis.* Only *B. pfeifferi* snails collected from Buluqute stream shed schistosome cercariae while the *B. pfeifferi* collected from Yamo stream did not.

### Mice infection

Eggs of *S. mansoni* were detected in feces of infected mice on day 40 post exposure. The adult worms from each of the sacrificed mouse were counted and gender was determined. A total of 311 male, 272 female, and 62 copula worms were harvested from the mesenteric veins.

## Discussion

Understanding epidemiological characteristics of intestinal schistosomiasis is important to inform control strategies in areas where poor hygienic standards, ideal environmental and climatic as well as malacological conditions favor the transmission of the parasites. The present study assessed the transmission and prevalence of *S. mansoni* infections in Yachi areas, Jimma Zone, southwestern Ethiopia.

The prevalence of *S. mansoni* infection observed in the present study was 42.9%. This agrees with the findings of studies conducted among school age children in Ziway Dugida Bora District (43.2%) [14] and Hayk area (45%) [15]. However, the present study revealed a higher prevalence rate of *S. mansoni* compared to studies conducted in other parts of Ethiopia (5.95%) [16], Kenya (16.5%) [17], Uganda (10.7%) [18] and Nigeria (12.1%) [19]. These variations might be attributed to differences in water contact activities, ecological features and proximity to cercariae-infested water bodies in the study areas. The high prevalence of *S. mansoni* infection in the present study might also be attributable to the socioeconomic problems such as poor personal hygiene and lack of clean water supply in the area.

The prevalence and intensity of *S. mansoni* infection in the present study were higher among males compared to females and this is in agreement with similar studies from other parts of Ethiopia [20], Brazil [21], Nigeria [22] and Sudan [23]. However, in Ghana [24], a significantly lower prevalence and infection intensity were observed among males compared to female children. The higher prevalence and intensity observed among male children in this study could be attributed to the frequent water contact behavior of male compared to female children. Male children more likely engage in recreational activities including bathing, swimming and playing in cercariae-infested water; hence they are more frequently exposed to infective water bodies than females. Moreover, the male children living in study area usually participate in small-scale irrigated agriculture which would also increase the chance of exposure to cercaria containing water.

The prevalence and intensity of *S. mansoni* infection were significantly higher among children attending Yachi Yisa School than those who attend Yachi Efo School. The higher prevalence and intensity schistosomiasis in Yachi Yisa School could be attributed to the presence of cercariae- infested water bodies close to the school that increases the probability of continuous transmission of the disease to the children having longer and frequent contact with the water. This agrees with the study done in Malawi [25] which indicated a higher infection rate in children who study in the school close to water bodies than those who studied the school away from the water sources.

Our findings show that almost 12% of *S. mansoni* infections were heavy intensity and nearly 11% of the infections were of moderate intensity. The higher percentages of heavy and moderate intensity *S. mansoni* infections observed in the present study is in line with previous studies [15, 26]. The high percentage of heavy infection intensity revealed in the present study is likely to cause morbidity in children infected with *S. mansoni* as the complications and clinical manifestations are associated with the intensity of infection. This suggests that school based mass drug administration (MDA) with praziquantel is needed to reduce morbidity and interrupt the transmission of schistosomiasis in the area.

Malacological surveys are useful for understanding the distribution and population dynamics of potential snails which play key roles in the development and transmission of schistosomes. Snail survey in the area showed that Buluqute Stream, which is close to Yachi Yisa Elementary School harbored infected *B. pfeifferi* snails. This stream had dense vegetation, algae, and muddy bottoms and the water was turbid and slow flowing (rate of 0.28 m/second). Additionally, restriction of water movement for irrigation purpose has created suitable ecology for the breeding of snail intermediate hosts and transmission of schistosomiasis. More importantly, collection of schistosome-infected *B. pfeifferi* in water bodies and harvesting of adult worms in the blood vessels of lab-infected mice confirm the establishment of transmission of *S. mansoni* in the study area.

Our results also show that 70.4% of the school children were infected with at least one species of intestinal helminth parasite. The high prevalence of helminth infection is consistent with the findings reported in different areas of the country, 71.3% in Lake Tana Basin [27], 76.7% in Mizan-Aman Town [28], and 73.7% in southern Ethiopia [29]. However, the prevalence of helminth infection obtained in the present study was higher than reports in central [30] and northwestern Ethiopia [31] which reported prevalence of 35.5 and 22.7%, respectively. The relatively higher prevalence of helminth infection observed in our study compared to the other studies may be attributed to inadequate sanitary conditions and lack of clean water for drinking and domestic activities. The absence of preventive control in the area could also be the reason for the high prevalence of the disease in this study.

A higher prevalence of intestinal helminth infection with any species was obtained using both methods compared to results estimated via either methods alone. On the other hand, KK thick smear technique revealed a higher sensitivity than the FECT in detecting *S. mansoni*, *T. trichiura*, *A. lumbricoides*, *Fasciola* species, *E. vermicularis*, and *S. stercoralis*, while the opposite was observed for *H. nana* and *Taenia* species. However, the use of FECT in the present study did not increase the prevalence of *S. mansoni* infection as all cases diagnosed by FECT were also found to be positive by KK thick smear technique. Besides the low intensity of *S. mansoni* infection obtained in this study, the sieving step in KK smear preparation allowing more filtered stool to be examined might be the reason for the higher prevalence rate obtained with KK thick smear technique compared to the FECT. Therefore, employing the KK thick smear technique and the FECT together are suggested for studying the epidemiology of intestinal helminth infection among children in areas where multiple parasites are found.

Although the current study was carefully conducted, we are aware of its limitations. First, the parasitological survey was conducted in few schools with similar ecology and environmental conditions. Given the focal epidemiology of schistosomiasis based on climatic conditions and the distribution of snail hosts, the current findings will not be inferred to other schools found in different settings. Secondly, the study did not assess the key factors that expose the children to schistosomiasis (e.g. sanitation, water contact behaviour and parent’s level of education), and further community-based studies are needed to identify potential risk factors associted with transmission of the disease in the area.

## Conclusion

The observation of field-caught *B. pfeifferi* snails shedding cercariae and establishment of *S.mansoni* infection in laboratory bred mice confirmed the transmission of intestinal schistosomiasis in Yachi areas, southwestern Ethiopia. Moreover, the study revealed that a moderate prevalence of *S. mansoni* infection was observed among school children in the study area. Besides MDA with praziquantel for schistosomiasis, sustained MDA of other intestinal helminths are also indicated. Additionally, other approaches including snail control for schistosomiasis and non-specific ones such as improved sanitation and provision of safe water are recommended to complement MDA for all intestinal worms.

## Additional file

Additional file 1: Multilingual abstracts in the five official working languages of the United Nations. (PDF 325 kb)

## Abbreviations

ARMEC: Arithmetic mean of egg count
*CI*: Confidence interval
EPG: Egg per gram
ERB: Ethical Review Board
FECT: Formol-ether concentration technique
KK: Kato-Katz
MDA: Mass drug administration
SPSS: Statistical package of social science
SSA: Sub-Saharan Africa
STH: Soil transmitted helminth
WHO: World Health Organization
