# Supplementary material for: Transmission of Schistosoma mansoni in Yachi areas, southwestern Ethiopia: new foci
Source: Infect Dis Poverty. 2019 Jan 10;8:1. doi: 10.1186/s40249-018-0513-5 (PMC6327402; doi:10.1186/s40249-018-0513-5)
Supplement: Supplementary file 1 — Multilingual abstracts in the five official working languages of the United Nations. (PDF 325 kb) [file 40249_2018_513_MOESM1_ESM.pdf]

Translation of the abstract into the five official working languages of the United Nations

انتقال مرض *البلهارسيا المانسونية* في مناطق ياتشي ، جنوب غرب إثيوبيا: بؤر جديدة

تيشومى بيكانا، وى هو، سونغ ليانغ، بيرهانو إركو

الملخص

الخلفية: *داء البلهارسيات المعوي الناجم عن البلهارسيا المانسونية* هو مرض واسع الانتشار في إثيوبيا؛ حيث يتم الإبلاغ عن ظهور بؤر جديدة للإصابة بالمرض باستمرار في ما يلي تقرير عن مواقع الإصابة بالبلهارسيا المانسونية S.mansoni الجديدة ومعدل انتشارها بين أطفال المدارس في مناطق ياتشى بجنوب غرب إثيوبيا.

المنهجية: أجريت دراسة مستعرضة بين تلاميذ المدارس الابتدائية في مناطق بيسا ياتشى وافو ياتشى بجنوب غرب إثيوبيا، في الفترة من نيسان/أبريل 2017 إلى يونيو/حزيران 2017. ثلاثة مائة سبعة عشر تلميذاً تتراوح أعمارهم بين 6 و 15 سنة اختيروا عشوائياً لتوفير عينات البراز لفحص إصابتهم بالداء الديداني بالاستعانة بتقنية كاتو-كاتز وتقنية الفورمول والأثير لتركيز الطفيليات. كما أُجري مسحاً لعدد الحلزونات لتقييم إمكانية وجود عدوى البلهارسيا المانسونية لدى حلزون المياه العذبة Biomphalaria pfeifferi. كذلك، تم تعريض الفئران في المختبرات إلى طور السركاريا المذنبة من البلهارسيا المانسونية الناتج عن الحلزون Biomphalaria pfeifferi جماعياً لتحديد نوعية البلهارسيا المانسونية بشكل قاطع.

النتائج: من بين عينات البراز التي جرى فحصها باستخدام لطخة كاتو-كاتز سميكة مزدوجة وتقنية الفورمول والاثير لتركيز الطفيليات مرة واحدة، والتي بلغ عددها 317 عينة، جاءت نتائج 224 عينة (70.7%) إيجابية العدوى بنوع واحد على الأقل من طفيليات الداء الديداني. كان الطفيلي الأكثر انتشاراً هو *البلهارسيا المانسونية* (42.9%) تليها الدودة السوطية (34.1%) والصفر الخراطيني (14.2%). وكان معدل انتشار عدوى *البلهارسيا المانسونية أعلى بكثير بين أطفال مدرسة بيسا ياتشي (49.4%) منه بين أطفال مدرسة إيفو ياتشي (35.6%) (القيمة الاحتمالية=* 0.002). الدراسة اكتشفت أيضاً انتشارًا أعلى بكثير لعدوى البلهارسيا المانسونية بين الذكور *(51.2٪) عن الإناث (33.1٪) (القيمة الاحتمالية* <0.001). *ومع ذلك،،* فإن معدل انتشار عدوى البلهارسيا المانسونية لم يكن مرتبطاً إلى حد كبير بالفئات العمرية (*القيمة الاحتمالية* = 0.839). *حلزون Biomphalaria pfeifferi* المصاب بالبلهارسيا *تم جمعه من المسطحات المائية الموجودة في المنطقة محل الدراسة.* كما جُمعت ديدان بلهارسيا مانسونية بالغة من الأوردة المساريقية لفئران التجارب بالمختبر بعد مرور ستة أسابيع من تعرّضها للعدوى.

الاستنتاجات: كشفت الدراسة عن ظهور بؤر جديدة للإصابة بعدوىالبلهارسيا المانسونية وكذلك عن وجود معدل انتشار متوسط لمرض البلهارسيا في مناطق ياتشى. ومن ثم، يوصي بعلاج جميع الأطفال في سن المدرسة مرة كل سنتين. يجب تكملة العلاج الجماعي بدواء برازيكونتيل بأساليب مكافحة الحلزون وأساليب المكافحة غير المحددة، والتي تتضمن توفير موارد المياه النظيفة والتوعية الصحية

Translated from English version into Arabic by Heba Kandel and Mais Salsa, through

TRANSLATORS
WITHOUT BORDERS

曼氏血吸虫在埃塞俄比亚西南部 Yachi 地区的一个新疫源地

Teshome Bekana, Wei Hu, Song Liang and Berhanu Erko

摘要：

引言：曼氏血吸虫感染可造成肠道血吸虫病，该病在埃塞俄比亚地区分布广泛，而且不断增加新的疫源地。在埃塞俄比亚西南部Yachi地区，本研究发现了一个新的曼氏血吸虫传播点，并在该地区调查学龄儿童中曼氏血吸虫感染的流行情况。

方法：2017年4至6月，在埃塞俄比亚西南部的Yachi Yisa和Yachi Efo 地区的两个小学的学生中开展了横断面调查研究，随机挑选了317名6–15岁内的学生，对其提供的粪便样本进行病原学检查（Kato-Katz法和甲醛乙醚浓度测定法），判断其蠕虫感染情况。检测菲氏双脐螺以

评估血吸虫感染情况。把实验室饲养小鼠暴露于菲氏双脐螺逸出的尾蚴以被感染，进而鉴别血吸虫虫种。

结果：本研究分别采用Kato-Katz厚涂片法（两次）和甲醛乙醚浓度测定法对317份粪便样本进行检测。结果发现，有224例（70.7%）至少感染一种肠道蠕虫，最常见的感染虫种是曼氏血吸虫(42.9%)，其次是毛首鞭形线虫(34.1%)和蛔虫(14.2%)。Yachi Yisa 小学被调查学生中的曼氏血吸虫流行率 (49.4%)明显高于Yachi Efo 小学 (35.6%)(P = 0.002)。男童中的曼氏血吸虫感染的流行率(51.2%)明显高于女童(33.1%) (P < 0.001)。但是，曼氏血吸虫感染的流行率和人群年龄组别没有明显的相关性(P = 0.839)。此外，在研究地区的水域中收集到感染血吸虫的菲氏双脐螺。实验小鼠暴露六周后，在其门静脉中收集到曼氏血吸虫的成虫。

结论：本研究确定了一个曼氏血吸虫感染的新传播疫源地，Yachi地区为血吸虫病中度流行区。因此，我们建议对所有学龄儿童进行两年一次血吸虫病治疗。开展吡喹酮群体治疗，加强螺的管理控制，并实施一些非具体性的控制措施包括提供清洁水源，健康教育等。

Translated from English version into Chinese by Xin-Yu Feng, edited by Jin Chen

CHINESE CENTER FOR DISEASE CONTROL AND PREVENTION
NATIONAL INSTITUTE OF PARASITIC DISEASES

Transmission du *Schistosoma mansoni* dans les régions de Yachi, sud-ouest de l'Éthiopie: nouveaux foyers

Teshome Bekana, Wei Hu, Song Liang et Berhanu Erko

Résumé

Contexte: *Le Schistosoma mansoni*, qui cause la schistosomiase intestinale, est largement répandu en Éthiopie et de nouveaux foyers de transmission sont continuellement signalés. Nous rapportons ici les nouveaux sites de transmission et la prévalence de l'infection au *S.mansoni* chez les écoliers des régions de Yachi, au sud-ouest de l'Éthiopie.

Méthodes: Une enquête transversale a été menée auprès des élèves des écoles primaires de Yachi Yisa et Yachi Efo, au sud-ouest de l'Éthiopie, d'avril à juin 2017. Trois cent dix-sept élèves âgés entre six et 15 ans ont été choisis aléatoirement pour fournir des échantillons de selles en vue de détecter l'infection par les helminthes en utilisant les techniques Kato-Katz et de concentration formol-éther. Une étude sur les escargots a été menée afin d'évaluer l'infection par le schistosome dans les *Biomphalaria pfeifferi*.Des souris de laboratoire ont été exposées aux cercaires de schistosome produits par des*B. pfeifferi en masse* afin d'identifier de manière définitive les espèce de *Schistosoma.*

Résultats: Sur les 317 échantillons de selles examinés en utilisant un double frottis épais de Kato-Klatz et une concentration formol-éther simple, 224 (70.7%) étaient positifs pour au moins une espèce d'helminthes intestinaux. Le parasite le plus répandu était le*S. mansoni* (42.9%) suivi par le *Trichuris trichiura* (34.1%) et *l'Ascaris lumbricoides* (14.2%). La prévalence de l'infection au *S. mansoni* était significativement plus élevée parmi les élèves de l'école de Yachi Yisa (49.4%) par rapport à ceux de l'école de Yachi Efo (35.6%) (P = 0.002). L'étude a également révélé qu'il y avait une prévalence bien plus élevée de l'infection au*S.mansoni* chez les hommes (51.2%) que chez les femmes (33.1%) (P < 0.001). Toutefois , la prévalence de l'infection au *S.mansoni* n'était pas associée de façon significative aux catégories d'âge (P = 0.839). Les escargots*B. pfeifferi* infectés par les schistosomiases ont été récoltés dans les étendues d'eau de la région où l'étude a été menée. Après six semaines post-exposition, les vers adultes*S. mansoni* ont été récoltés des veines mésentériques des souris de laboratoire.

Conclusions: L'étude a montré l'établissement de nouveaux foyers de transmission du *S. mansoni* et la prévalence modérée de la schistosomiase dans les régions de Yachi. Il est donc recommandé de traiter tous les enfants d'âge scolaire tous les deux ans. Les approches en matière de contrôle des mollusques et de contrôle non spécifique comprenant l'approvisionnement en eau potable et aux services de l'éducation de santé devraient également contribuer à la distribution massive du praziquantel.

Translated from English version into French by Isabelle Redon and Marie Piaget, through

TRANSLATORS WITHOUT BORDERS

Заражение *шистосомой Мансони* в районах Ячи юго-западной Эфиопии: новые очаги

Тешом Бекана, Вэй Ху, Сун Лян и Берхану Эрко

Аннотация

Справочная информация: *шистосома Мансони*, возбудитель кишечного шистосомоза, широко распространена в Эфиопии, более того, продолжают поступать сообщения о новых очагах передачи. В данном документе сообщается о новых очагах инфицированности и заболеваемости *шистосомой Мансони* среди детей школьного возраста в районах Ячи юго-западной Эфиопии.

Методы: С апреля 2017 г. по июнь 2017 г. было проведено перекрёстное исследование среди детей начального школьного возраста, обучавшихся в школах Yachi Yisa и Yachi Efo на юго-западе Эфиопии. Триста семнадцать школьников в возрасте от шести до 15 лет были отобраны методом случайной выборки для предоставления образцов анализа кала для диагностики заражения гельминтами методами Като-Каца и формалин-эфирной концентрации. Было проведено исследование на улитках для изучения заражения шистомозом улиток вида *Biomphalaria pfeifferi.* Лабораторные мыши также были заражены церкариями шистосом, переносимыми улитками вида *B. pfeifferi* , *поголовно* для точной идентификации видов *шистосомы*.

Результаты: Из 317 образцов кала, исследованных методом Като-Каца с использованием мазка двойной толщины и однократной формалин-эфирной концентрации, было выявлено, что 224 (70,7 %) образца были положительными по меньшей мере для одного вида кишечного гельминта. Наиболее распространенными паразитами оказались *шистосома Мансони* (42,9 %), за ними следовали *власоглав* (34,1 %) и *человеческая аскарида* (14,2 %). Распространенность заражения *шистосомой Мансони* была значительно выше у детей, посещавших школу Yachi Yisa (49,4 %), чем у тех, кто ходил в школу Yachi Efo (35,6 %) (P = 0,002). В данном исследовании также было определено, что заболеваемость *шистосомой Мансони* значительно более распространена среди мужчин (51,2 %), чем среди женщин (33,1 %) (P < 0,001). Однако связь между заболеваемостью*шистосомой Мансони* и возрастными категориями была незначительной (n = 0,839). *Улитки вида* B. pfeifferi, зараженные шистосомами, были собраны из водоёмов, обнаруженных в районе исследования. Через шесть недель после заражения, взрослые черви *шистосомы Мансони* были собраны из мезентериальных вен разводимых в лаборатории мышей.

Выводы: В ходе исследования были обнаружены новые очаги заражения*шистосомой Мансони* и умеренная заболеваемость шистосомозом в районах Ячи. Исходя из этого, раз в

два года рекомендуется проводить лечение всех детей школьного возраста. Методы как контроля, так и неспецифического контроля за улитками, включая снабжение чистой водой и санитарное образование, следует также совмещать с массовым применением празиквантела.

Translated from English version into Russian by Alexander Vareiko and Liudmila Tomanek, through

Transmisión del *Schistosoma mansoni* en áreas Yachi del sudoeste de Etiopía: nuevos focos

Teshome Bekana, Wei Hu, Song Liang and Berhanu Erko

Resumen

Contexto: *El parásito Schistosoma mansoni*, que causa esquistosomiasis intestinal, está ampliamente diseminado en Etiopía, y se reportan de manera constante nuevos focos de transmisión. Aquí informamos de nuevos sitios de transmisión y sobre la prevalencia de la infección causada por el *S.mansoni* entre niños en edad escolar en las áreas de Yachi en el sudoeste de Etiopía.

Métodos: Se realizó una encuesta transversal entre alumnos de escuelas primarias de Yachi Yisa y Yachi Efo, en el sudoeste de Etiopía, desde abril de 2017 hasta junio de 2017. Se seleccionaron al azar trescientos diecisiete niños entre 6 y 15 años para tomar muestras de heces para el examen de infección por helmintos mediante las técnicas de Kato-Katz y de concentración de formol-éter. Se realizó un estudio sobre caracoles para evaluar la infección por esquistosoma en los *Biomphalaria pfeifferi.* Los ratones criados en laboratorio también fueron expuestos a cercarias esquistosómicas emitidas por*B. pfeifferi en masa* para la identificación definitiva de las especies de *Schistosoma*.

Resultados: De las 317 muestras de heces examinadas usando dobles frotis de Kato-Katz y técnicas únicas de concentración de formol-éter, 224 (70,7%) resultaron positivas para al menos una especie de helmintos intestinales. El parásito dominante fue *S. mansoni* (42,9%) seguido de *Trichuris trichiura* (34,1%) y *Ascaris lumbricoides* (14,2%). La prevalencia de la infección por el *S. mansoni* fue significativamente más alta entre los niños que asisten a la escuela Yachi Yisa (49,4%) que en la escuela Yachi Efo (35,6%) (P = 0,002). El estudio también reveló que hubo una prevalencia significativamente más alta de la infección por *S.mansoni* entre los varones (51.2%) que entre las mujeres (33,1%) (P <0,001). Sin embargo, la permanencia de la infección por*S.mansoni* no se asoció significativamente con las categorías por edad (P = 0,839). *Los caracoles* B. pfeifferi infectados con esquistosomas fueron recolectados de los cuerpos de agua encontrados en el área de estudio. Después de las seis semanas posteriores a la exposición, se cosecharon gusanos adultos *S. mansoni* de las venas mesentéricas de ratones criados en laboratorio.

CONCLUSIONES: El estudio reveló la creación de nuevos focos de transmisión de *S. mansoni* y una prevalencia moderada de esquistosomiasis en las áreas de Yachi. Por lo tanto, se recomienda el tratamiento de los niños en edad escolar una vez cada dos años. El control de los caracoles y los enfoques de control no específicos, entre los cuales se incluye la provisión de suministros de agua limpia y la educación sanitaria, también deberían complementar la administración masiva de praziquantel.

Translated from English version into Spanish by Guadalupe Barua and E Passadore, through

TRANSLATORS
WITHOUT BORDERS
